# Supplementary material for: Mutations in PCYT2 disrupt etherlipid biosynthesis and cause a complex hereditary spastic paraplegia
Source: Brain. 2019 Oct 22;142(11):3382–97. doi: 10.1093/brain/awz291 (PMC6821184; doi:10.1093/brain/awz291)
Supplement: awz291_Supplementary_Data [file awz291_supplementary_data.zip › awz291-Suppl_data/Supplementary_Data4.zip!/Supplementary information 4 - plasma lipidomics.zip!/Plasma lipidomics/Control vs Patient3b/Statistical plots and data/PE[O] species/Heatmap.pdf]

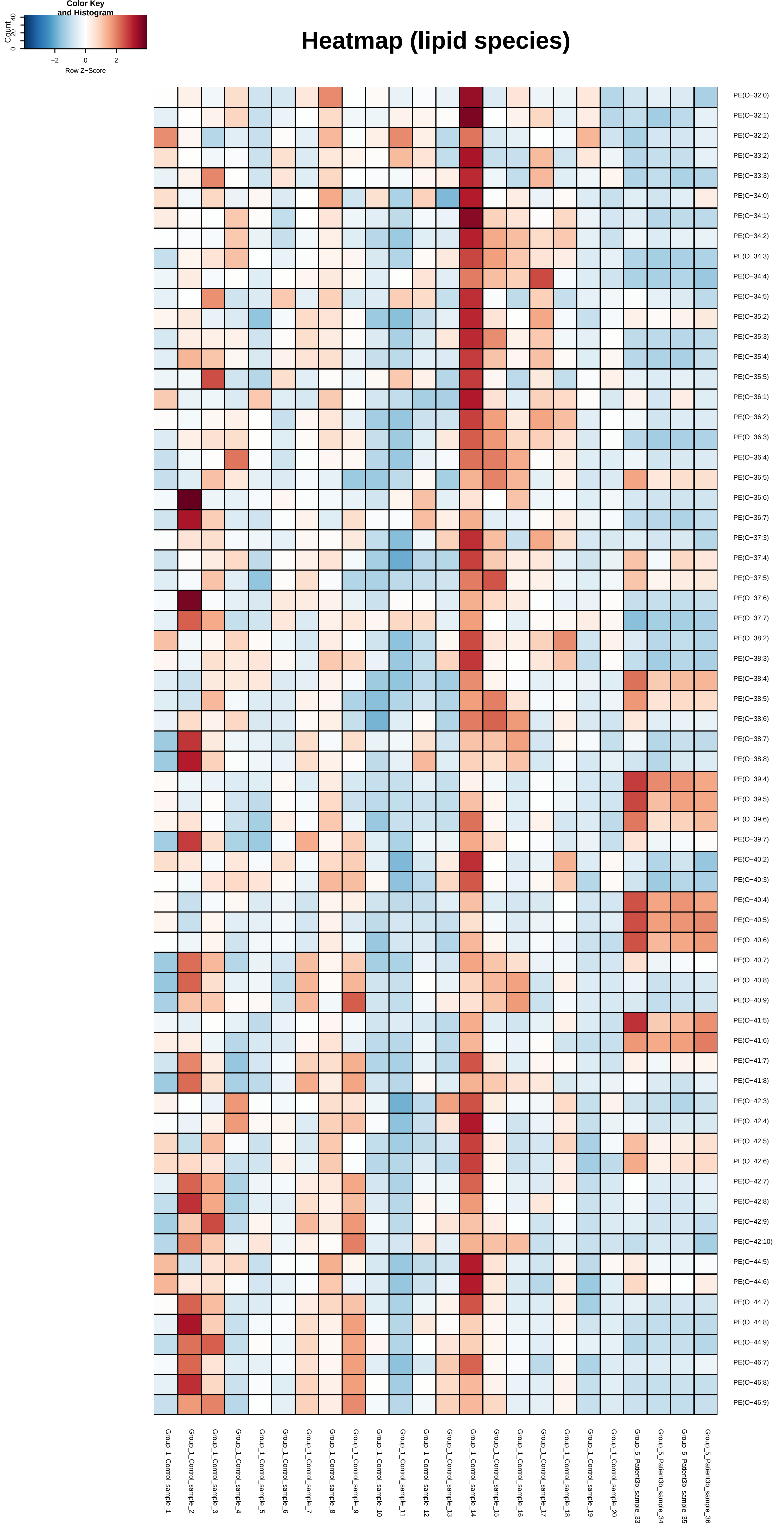
Heatmap (lipid species)
Color Key and Histogram
Count
0
20
40
−2
0
2
Row Z−Score
PE(O−32:0)
PE(O−32:1)
PE(O−32:2)
PE(O−33:2)
PE(O−33:3)
PE(O−34:0)
PE(O−34:1)
PE(O−34:2)
PE(O−34:3)
PE(O−34:4)
PE(O−34:5)
PE(O−35:2)
PE(O−35:3)
PE(O−35:4)
PE(O−35:5)
PE(O−36:1)
PE(O−36:2)
PE(O−36:3)
PE(O−36:4)
PE(O−36:5)
PE(O−36:6)
PE(O−36:7)
PE(O−37:3)
PE(O−37:4)
PE(O−37:5)
PE(O−37:6)
PE(O−37:7)
PE(O−38:2)
PE(O−38:3)
PE(O−38:4)
PE(O−38:5)
PE(O−38:6)
PE(O−38:7)
PE(O−38:8)
PE(O−39:4)
PE(O−39:5)
PE(O−39:6)
PE(O−39:7)
PE(O−40:2)
PE(O−40:3)
PE(O−40:4)
PE(O−40:5)
PE(O−40:6)
PE(O−40:7)
PE(O−40:8)
PE(O−40:9)
PE(O−41:5)
PE(O−41:6)
PE(O−41:7)
PE(O−41:8)
PE(O−42:3)
PE(O−42:4)
PE(O−42:5)
PE(O−42:6)
PE(O−42:7)
PE(O−42:8)
PE(O−42:9)
PE(O−42:10)
PE(O−44:5)
PE(O−44:6)
PE(O−44:7)
PE(O−44:8)
PE(O−44:9)
PE(O−46:7)
PE(O−46:8)
PE(O−46:9)
Group_1_Control_sample_1
Group_1_Control_sample_2
Group_1_Control_sample_3
Group_1_Control_sample_4
Group_1_Control_sample_5
Group_1_Control_sample_6
Group_1_Control_sample_7
Group_1_Control_sample_8
Group_1_Control_sample_9
Group_1_Control_sample_10
Group_1_Control_sample_11
Group_1_Control_sample_12
Group_1_Control_sample_13
Group_1_Control_sample_14
Group_1_Control_sample_15
Group_1_Control_sample_16
Group_1_Control_sample_17
Group_1_Control_sample_18
Group_1_Control_sample_19
Group_1_Control_sample_20
Group_5_Patient3b_sample_33
Group_5_Patient3b_sample_34
Group_5_Patient3b_sample_35
Group_5_Patient3b_sample_36
